# Genome Sequence of *Sinorhizobium meliloti* Rm41

**DOI:** 10.1128/genomeA.00013-12

**Published:** 2013-01-15

**Authors:** Stefan Weidner, Birgit Baumgarth, Michael Göttfert, Sebastian Jaenicke, Alfred Pühler, Susanne Schneiker-Bekel, Javier Serrania, Rafael Szczepanowski, Anke Becker

**Affiliations:** a Centrum für Biotechnologie (CeBiTec), Universität Bielefeld, Bielefeld; b Institut für Genetik, Technische Universität Dresden, Dresden; c LOEWE-Zentrum für Synthetische Mikrobiologie, Philipps-Universität Marburg, Marburg, Germany

## Abstract

*Sinorhizobium meliloti* Rm41 nodulates alfalfa plants, forming indeterminate type nodules. It is characterized by a strain-specific K-antigen able to replace exopolysaccharides in promotion of nodule invasion. We present the Rm41 genome, composed of one chromosome, the chromid pSymB, the megaplasmid pSymA, and the nonsymbiotic plasmid pRme41a.

## GENOME ANNOUNCEMENT

A peculiar feature of indeterminate nodulation is the general requirement for bacterial exopolysaccharides (EPS) for successful nodule invasion (1). The first exception was reported for *Sinorhizobium meliloti* AK631, an EPS-deficient derivative of strain Rm41. Rm41 and the parental strain of *S. meliloti* 1021, SU47, are different natural isolates from Hungary and Australia, respectively. Strain Rm41 was originally obtained from a screen for lysogenic strains isolated from root nodules of *Melilotus* and *Medicago* (2). A circular genetic map of its chromosome was published in 1977 (3). Although AK631 is unable to produce EPS, it was fully infective on *Medicago sativa* as a result of the strain-specific K_R_5 antigen, which functionally can replace EPS in promotion of the invasion process (4). Further differences between the reference strain Rm1021 and Rm41 comprise restriction systems apparent in different phage susceptibility profiles (5), the ability of Rm41 to catabolize calystegines contributing to colonization of the rhizosphere of nonlegume plants (6), and the presence of a second quorum-sensing system in Rm41 (7). The latter two properties could be attributed to the presence of the nonsymbiotic plasmid pRme41a. The genome sequence of Rm41 will reveal further differences, detailed information about the genetic basis of symbiotically active strain-specific surface polysaccharides, and the gene load of the accessory plasmid pRme41a.

For whole-genome sequencing, a combination of shotgun (GS FLX titanium) and long paired-end (GS FLX standard) sequencing using the GS FLX system (Roche Diagnostics) produced a 34-fold genome coverage. Read assembly with the Newbler software (Roche Diagnostics) resulted in 22 scaffolds and 201 contigs. For gap closure and finishing, sequences (ABI 3730xl DNA analyzer) of 256 PCR products were acquired. Using the Consed software package (8), the complete genome could be assembled into a chromosome (replicon size/G+C content/number of protein coding genes: 3,679,105 bp/62.75%/3,499), two symbiotic plasmids (pSymA: 1,559,666 bp/60.57%/1,589; and pSymB: 1,664,896 bp/62.33%/1,519), and the nonsymbiotic plasmid pRme41a (246,023 bp/59.33%/237), resulting in a total size of 7,149,690 bp. Automated annotation of the genome was performed using GenDB (9). For gene products with bidirectional best blast hits with ≥90% identity, the annotation of *S. meliloti* 1021 (10) was transferred to *S. meliloti* Rm41, which was applicable to 1,583 predicted genes. The genome contains 54 tRNA genes and 3 *rrn* loci mapping to the chromosome and an additional tRNA gene on pSymB.

### Nucleotide sequence accession numbers.

The nucleotide genome sequence of *S. meliloti* Rm41 has been deposited in the EMBL Nucleotide Sequence Database (EMBL-Bank) under accession numbers HE995405 to HE995408.
